# A Randomized Controlled Trial of an SMS‐Based Brief Contact Intervention for People Bereaved by Suicide

**DOI:** 10.1111/sltb.70043

**Published:** 2025-09-03

**Authors:** Katie McGill, Navjot Bhullar, Alayna Carrandi, Philip J. Batterham, Sarah Wayland, Myfanwy Maple

**Affiliations:** ^1^ MH‐READ, Hunter New England Local Health District Newcastle Australia; ^2^ University of Newcastle Newcastle Australia; ^3^ University of New England Armidale Australia; ^4^ Health Research Institute University of Canberra Canberra Australia; ^5^ Australian and New Zealand Intensive Care Research Centre, School of Public Health and Preventative Medicine Monash University Melbourne Australia; ^6^ Australian National University Canberra Australia; ^7^ Central Queensland University Sydney Australia

## Abstract

**Introduction:**

Brief contact interventions (BCI) refer to short messages delivered proactively to a specific target population. The aim of this study was to test the effectiveness of a mobile phone short‐message service (SMS) BCI for people bereaved by suicide.

**Methods:**

Participants were randomly allocated. The BCI group received text messages over a 6‐week period. The active control group received the intervention website. Pre‐ and post‐intervention surveys assessed demographic, suicide exposure and five key outcomes (psychological distress, suicidal ideation, complicated grief, resilience, and professional help‐seeking intentions). BCI participants were also invited to participate in an interview post‐intervention.

**Results:**

Of 99 participants randomized, 52 BCI and 47 control completed pre‐intervention surveys. Post‐intervention response rates were low (BCI: *n* = 15; 28.85%; active control: *n* = 16; 34.04%), with no statistically significant changes in key outcome measures. Eight BCI participants completed follow‐up interviews. Relevance, timing of support, benefit to bereavement, and recommendations for scaling were identified.

**Conclusions:**

Recruitment and retention challenges meant the effectiveness of the BCI could not be statistically determined. Qualitative evidence supported BCI acceptability for people bereaved by suicide. Recommendations to improve the intervention include embedding the BCI within existing postvention services offered soon after a death occurs and tailoring of messages to individuals' needs.

**Trial Registration:**

This trial was registered with the Australian New Zealand Clinical Trial Register (ACTRN12621001430820)

## 1 Introduction

Globally, 1 in 5 people have been exposed to, or bereaved by, suicide during their lifetime (Andriessen et al. 2017). In Australia, the prevalence of lifetime exposure to suicide is considerably higher—58% (Maple et al. 2019) including 38% for close relationships (ABS 2022). There are many significant health consequences of suicide exposure among relatives (Pitman et al. 2017), friends, and acquaintances (Maple, Cerel, et al. 2017) of the deceased, including poorer physical health (Erlangsen et al. 2017; Spillane et al. 2018) and mental health (Pitman et al. 2014), and greater risk for future suicide and suicide attempts (Hill et al. 2020). While addressing the needs of those bereaved by suicide is identified as a key priority by the World Health Organization (2014), current large‐scale suicide prevention efforts in Australia have had limited focus on those bereaved by suicide. High‐quality evidence for the effectiveness of postvention interventions, after the loss of someone to suicide, is lacking (Andriessen, Krysinska, Hill, et al. 2019).

Postvention interventions vary and include face‐to‐face therapy (e.g., cognitive behavior therapy and supportive counseling), group and family interventions, individual writing interventions, and peer support approaches (Andriessen, Krysinska, Hill, et al. 2019; Linde et al. 2017) in a variety of private, community, and school‐based settings. Key components thought to underlie the effectiveness of postvention interventions include structured approaches that strengthen access to available supports (Hofmann et al. 2025). Digital interventions to deliver bereavement support (including for suicide bereavement) have begun to garner interest (Lenferink et al. 2020; Galway et al. 2019; Wagner et al. 2020). In light of the call for proactive, targeted postvention responses (Andriessen, Krysinska, Hill, et al. 2019; Finlayson‐Short et al. 2020), brief contact interventions (BCIs) delivered via technology that link people to additional support are a viable option for addressing the immediate information and support needs of the suicide bereaved. While not all BCIs are delivered via technology, they comprise short messages that provide support and encouragement to a specific target population group over a specified period of time, and/or direct individuals to where additional help or information can be sought (Milner et al. 2015).

For suicide prevention, BCIs have been shown to be effective in reducing self‐harm among people who have made a suicide attempt (Milner et al. 2015; Doupnik et al. 2020; Luxton et al. 2013), and may have a role in providing information to people after a suicide death about the grief process, help‐seeking encouragement, and/or short‐term emotional support (McGill, Bhullar, Pearce, et al. 2022). Digital BCIs, such as web and mobile phone‐based interventions, have also been implemented among bereaved populations more broadly (as opposed to specifically suicide bereaved individuals). These interventions have delivered therapeutic support, psychoeducation, and self‐help resources to support coping, functioning, and recovery after a death (Wagner et al. 2020; Zuelke et al. 2021). There is promising evidence for the effectiveness of digital BCIs in treating symptoms of grief, depression, and posttraumatic stress after bereavement (Wagner et al. 2020; Zuelke et al. 2021). However, given the unique needs of those bereaved by suicide, including stigma and differential responses by community networks, the often unexpected nature of the death, and the range of emotions associated with making meaning of the self‐inflicted and intentional nature of the death (e.g., anger, guilt, abandonment, etc.) (Pitman et al. 2017; Hofmann et al. 2025; Shields et al. 2017) such interventions require testing within this population. Thus, there is a need for well‐designed and rigorous effectiveness studies of BCIs for suicide‐bereaved communities.

Given promising evidence for their effectiveness in suicide prevention and general bereavement, a digital BCI for people bereaved by suicide may be appropriate to deliver support after a suicide death, linking people quickly into services and reducing morbidity and mortality associated with suicide bereavement. The current study reports a randomized controlled trial (RCT) with the primary aim of testing the effectiveness and acceptability of a mobile phone‐based short message service (SMS) BCI for people bereaved by suicide and assessing the added benefit of delivering text messages over standard psychoeducation via evidence‐based website delivery.

## 2 Method

### 2.1 Study Design

This unblinded, parallel RCT was registered with the Australian New Zealand Clinical Trial Register (ACTRN12621001430820). Ethics approval was granted by the University of New England's Human Research Ethics Committee (HE21‐175). Trial reporting follows the CONSORT guidelines (Moher et al. 2010) and, where appropriate, the CONSORT‐EHEALTH guidelines for web‐based and mobile health interventions (Eysenbach 2011).

### 2.2 Experimental Manipulations

Participants were randomly allocated to either the BCI intervention group or the active control group in a 1:1 ratio using an automated allocation process within the Qualtrics platform. The intervention group received a series of seven automated text messages via their preferred mobile phone number over a 6‐week period with links to webpages containing additional content. The text messages were co‐designed with suicide‐bereaved people, postvention workers, and researchers, including the content, timing, and framing of the messages. In the first instance, this was undertaken via an international survey of those bereaved by suicide, those who support them, and postvention researchers (*n* = 18) to examine needs and timing of support matched to these needs (McGill, Bhullar, Batterham, et al. 2022). Following this, draft messages were created by the authors, which were tested in focus groups with suicide‐bereaved individuals (*n* = 11) participating in focus groups facilitated by KM, with participants remunerated for their time. Messages were subsequently revised following this round of feedback. The content of text messages included: (1) Grief after suicide; (2) Practical challenges associated with bereavement; (3) Navigating relationships after suicide; (4) Accessing crisis support; (5) Looking after yourself; (6) Accessing peer support; and (7) Accessing professional support. Embedded within the text messages were links to closed webpages. The webpages were hosted by Everymind (www.lifeinmind.com.au) and contained further information about the topic, self‐care, and links to available supports. Appendix A contains the text message content and linked resources are available from the corresponding author.

Meanwhile, the active control group participants received a link to a website containing the same psychoeducation content at the end of the T1 survey, and were invited to access the website throughout the duration of the intervention period. No text messages or follow‐up reminders were sent to access this information. Active control groups have been successfully used to test the effectiveness and feasibility of other web‐based bereavement interventions (Zuelke et al. 2021). For this study, the use of an active control group allowed assessment of the degree to which regular, proactive prompts with supportive information increase exposure to and benefit from psychoeducation materials. At the end of the T2 survey completion, BCI participants were invited to participate in qualitative interviews to share their experiences of the intervention.

### 2.3 Participant Eligibility Criteria

Eligibility for this study included: 18 years or over; bereaved by suicide in the last year (self‐identified); living in Australia; proficient in the English language; not currently suicidal (self‐reported); and access to a mobile phone. Participants who did not meet the inclusion criteria were directed to a page with readily accessible support resources. To detect a small to moderate difference (*d* = 0.4) between the intervention and active control groups with 90% power, a sample of 266 was required. To account for up to 50% attrition (based on prior similar projects) (Batterham et al. 2018; Wagner et al. 2022), our aim was to recruit 266 participants per condition, resulting in a total sample of 532.

**FIGURE 1:**
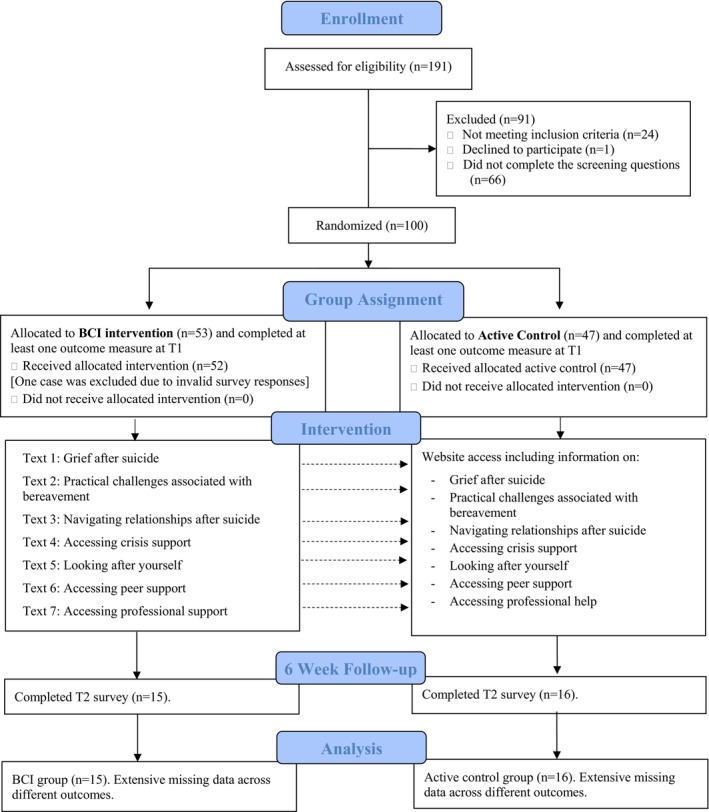
CONSORT participant flow chart.

As shown in Figure 1, of the 191 participants assessed for eligibility, 100 (52.36%) were randomly allocated to either a BCI group (*n* = 53) or an active control group (*n* = 47) (see Figure 1: CONSORT Participant flowchart). One case was excluded from the BCI group due to invalid responses at T1. Therefore, the final number of participants allocated to the BCI group at T1 was 52. Response rates at T2 for the BCI and active control groups were 28.85% (*n* = 15) and 34.04% (*n* = 16), respectively, representing an overall attrition rate of 68.69%.

The study sample predominantly comprised female respondents (92.9%), with an overall sample mean age of 47.32 years. Details of participant characteristics are noted in the Results section and summarized in Table 1.

**TABLE 1:** Summary of participant characteristics for experimental groups and total sample at T1.

	BCI group, *n* = 52	Active control group, *n* = 47	Total sample, *n* = 99
Demographics			
Age	**Mean (SD)**	**Mean (SD)**	**Mean (SD)**
Years of age	47.75 (13.63)	46.81 (13.67)	47.32 (13.58)
Missing: *n* (%)	1 (1.9%)	4 (8.5%)	5 (5.1%)
Gender	***n* (%)**	***n* (%)**	***n* (%)**
Male	5 (9.6%)	1 (2.1%)	6 (6.1%)
Female	46 (88.5%)	46 (97.9%)	92 (92.9%)
Prefer not to say	1 (1.9%)	—	1 (1.0%)
Country of birth	***n* (%)**	***n* (%)**	***n* (%)**
Australia	44 (84.6%)	40 (85.1%)	84 (84.8%)
Born overseas	8 (15.4%)	7 (14.9%)	15 (15.2%)
Language spoken at home	***n* (%)**	***n* (%)**	***n* (%)**
English	41 (78.8%)	38 (80.9%)	79 (79.8%)
Other	—	—	—
Missing	11 (21.2%)	9 (19.1%)	20 (20.2%)
Geographic location	***n* (%)**	***n* (%)**	***n* (%)**
Metropolitan	21 (40.4%)	22 (46.8%)	43 (43.4%)
Regional	21 (40.4%)	18 (38.3%)	39 (39.4%)
Rural	10 (19.2%)	6 (12.8%)	16 (16.2%)
Remote	—	1 (2.1%)	1 (1.0%)
Lived experience of suicide			
Relationship with the deceased^a^	***n* (%)**	***n* (%)**	***n* (%)**
Partner	6 (11.5%)	9 (19.1%)	15 (15.2%)
Child	13 (25.0%)	11 (23.4%)	24 (24.2%)
Parent	3 (5.8%)	3 (6.4%)	6 (6.1%)
Sibling	5 (9.6%)	3 (6.4%)	8 (8.1%)
Friend	9 (17.3%)	4 (8.5%)	13 (13.1%)
Client	1 (1.9%)	—	1 (1.0%)
Other	7 (13.5%)	4 (8.5%)	11 (11.1%)
Missing	8 (15.4%)	13 (27.7%)	21 (21.2%)
Self‐reported suicidality	***n* (%)**	***n* (%)**	***n* (%)**
Suicidal thoughts in last 12 months: Yes	28 (53.8%)	22 (46.8%)	50 (50.5%)
No	22 (42.3%)	20 (42.6%)	42 (42.4%)
Missing	2 (3.8%)	5 (10.6%)	7 (7.1%)
Suicide plan in last 12 months: Yes	10 (19.2%)	7 (14.9%)	17 (17.2%)
No	18 (34.65)	15 (31.9%)	33 (33.3%)
Missing	24 (46.2%)	25 (53.2%)	49 (49.5%)
Suicide attempt in last 12 months: Yes	1 (1.9%)	0 (0%)	1 (1.0%)
No	49 (94.2%)	41 (87.2%)	90 (90.9%)
Missing	2 (3.8%)	6 (12.8%)	8 (8.1%)
Suicide exposure			
Suicide exposure experience screener	**Mean (SD)**	**Mean (SD)**	**Mean (SD)**
Reported closeness to deceased	3.82 (1.24) (*n* = 44)	4.17 (1.12) (*n* = 35)	3.97 (1.20) (*n* = 79)
Missing: *n* (%)	8 (15.45)	12 (25.5%)	20 (20.2%)
Perceived impact of death	4.50 (1.00) (*n* = 44)	4.60 (0.81) (*n* = 35)	4.54 (0.92) (*n* = 79)
Missing: *n* (%)	8 (15.4%)	12 (25.5%)	20 (20.2%)
Cumulative exposure	**Mean (SD)**	**Mean (SD)**	**Mean (SD)**
Number of people they had known who had died by suicide	3.26 (2.80) (*n* = 43)	3.29 (3.42) (*n* = 35)	3.27 (3.07) (*n* = 78)
Missing: *n* (%)	9 (17.3%)	12 (25.5%)	21 (21.2%)

^a^ Participants could choose more than one response.

### 2.4 Recruitment and Procedure

After pausing the study at the onset of the COVID‐19 pandemic, participants were recruited using social media and professional network links from August 2021 until February 2022. Those interested in participating were directed to a webpage to complete eligibility screening questions. Information and relevant support services were offered, including immediate support options. After registering and providing digital consent, participants were randomly allocated to either a BCI group or an active control group, which was managed through the online Qualtrics platform. Following randomization, participants completed an online baseline (T1) survey. The T1 survey took approximately 30 min to complete and comprised a mix of standardized and tailored outcome measures and questionnaires. Six weeks after completing the T1 survey, participants were contacted via text message and asked to complete the follow‐up survey (T2), which comprised the same measures from T1 with additional questions exploring the acceptability, applicability, and practicality of the intervention for the BCI participants. Participants received up to four text message reminders to complete the T2 survey over a 2‐week period.

BCI participants were also invited to participate in an interview to share their experiences of the intervention and research. Interviews were conducted by authors [AC and KB]. Interviews were held via videoconferencing (Zoom) or phone and lasted approximately 30 min. Only audio was recorded for each interview, and the recording was transcribed; participants were provided an opportunity to review the transcript if they wished.

### 2.5 Measures

#### 2.5.1 Demographics

A range of sample characteristics was collected, including age, gender, geographic location; lived experience of suicide (assessed via relationship to the deceased; personal experience of suicidality in the last 12 months).

#### 2.5.2 Suicide Exposure

Information about *suicide exposure* was captured using two indices. First, through a 2‐item Suicide Exposure Experience Screener (SEES) (Maple et al. 2022) assessing reported closeness with the person who died by suicide and perceived impact of the suicide death. *Cumulative exposure* was assessed by asking respondents the number of people they had known who died by suicide.

### 2.6 Outcome Measures

*Psychological distress* was the primary outcome measure and was assessed using the Kessler‐10 (K10) (Kessler et al. 2003). It comprises 10 items assessed on a 5‐point Likert‐type scale ranging from 1 to 5, with 1 = *none of the time* and 5 = *all of the time*. Items are summed to create a composite score, with higher scores indicating greater psychological distress. The maximum score for K10 is 50, indicating severe distress, and the minimum score is 10, indicating no distress. The K10 had good internal consistency in this study (Cronbach *α* = 0.94 at T1).

Secondary outcomes were assessed as follows:

*Suicidal ideation* was measured using a 5‐item Suicidal Ideation Attributes Scale (SIDAS) (van Spijker et al. 2014). Items assess specific attributes of suicidal thoughts: frequency, controllability, and level of distress associated with the thoughts. The 11‐point Likert‐type scale ranges from 0 = *never/not close at all/not at all* to 10 = *always/full control/made an attempt*. One item is reverse‐scored before items are summed to create a composite score, with higher scores indicating greater severity of suicidal thoughts. Cronbach's α of 0.90 for SIDAS at T1 in this study was excellent.

*Complicated grief* was measured using a 19‐item Inventory of Complicated Grief (ICG) (Prigerson et al. 1995) that captures emotional distress specifically related to complicated grief. Items are rated on a 5‐point Likert scale ranging from 0 (not at all) to 4 (extremely), with higher scores indicating greater emotional distress due to bereavement grief. Items are summed to create a composite score of complicated grief. Participants with scores above 25 are considered at high risk for requiring clinical care. Cronbach's α of 0.92 for ICG at T1 in this study was excellent.

*Resilience* was measured using a 12‐item Resilience Appraisal Scale (RAS) (Johnson et al. 2010) which assesses respondents' appraisal of their ability to cope with emotions, solve problems, and seek social support. Items are rated on a 5‐point Likert scale ranging from 1 (strongly disagree) to 5 (strongly agree), with higher scores indicating greater resilience. Items are summed to create a composite overall resilience. A higher score indicates greater levels of resilience. Cronbach's *α* of 0.90 for the total RAS score at T1 in this study was excellent.

*Professional help‐seeking intentions* were measured using two items assessing help‐seeking intentions from formal sources in the General Help Seeking Questionnaire (GHSQ) (Prigerson et al. 1995). Items are assessed on a 7‐point Likert‐type scale (1 = Extremely unlikely to 7 = Extremely likely). Items are averaged, and higher scores indicate greater intention to seek help from mental health professionals (e.g., psychologist, social worker, counselor), doctors, or general practitioners. Cronbach's *α* of 0.67 for professional help‐seeking intentions in this study was less than what is considered acceptable (*α* = 0.70).

### 2.7 Manipulation Checks

#### 2.7.1 Intervention Feedback

Feedback and the BCI participants' experience of the intervention were explored through open‐ended questions in the T2 survey and through the qualitative interviews. Specifically, T2 survey included 15 statements about the intervention (e.g., “I enjoyed the intervention”), which participants rated for their level of agreement, and three open‐ended questions about what they liked and disliked about the intervention and how it could be improved. The qualitative interview explored participants' experiences of receiving the messages, their content and the linked web‐based materials.

#### 2.7.2 Webpage Traffic

Access to the webpages that the BCI linked to, and which all active control participants could also access, was monitored in aggregate for each experimental group. Specifically, the number of views and time spent on webpages was captured, with distinct URLs used for each experimental group to examine differences in traffic.

### 2.8 Data Analyses

Participants' data were included in the main analysis if they provided complete data for at least one measure at T1. Demographic and participant characteristics were compared across intervention groups using independent sample *t*‐tests. Mixed model analyses of variance (ANOVAs) were conducted using IBM SPSS Statistics software (v. 30.0.0.0 [172]) to examine group differences in key study outcomes from T1 to T2.

For the qualitative data (from the open‐ended survey questions and semi‐structured interviews), inductive analysis was used to understand the acceptability and experience of the intervention. Inductive coding involving a line‐by‐line analysis of responses, and following Braun and Clarke's (Braun and Clarke 2006) thematic analysis process, was performed by coders (primary coding‐ KB, AC; coding review‐ SW, MM) to explore the relevance of the intervention to participants' experiences, the timing of support, benefit to bereavement, and future recommendations for scaling up the intervention.

## 3 Results

### 3.1 Descriptive Statistics

#### 3.1.1 Participant Characteristics

A detailed summary of a range of participant characteristics is provided in Table 1. Of 78 participants who reported their relationship with the person who died by suicide, almost one quarter (24.2%) reported having lost a child, followed by those who reported losing a partner to suicide (15.2%).

Half of the participants (50.5%) reported suicidal ideation in the last 12 months, with one in six (17%) reporting a suicide plan and one participant reporting a suicide attempt in the last 12 months. On average, participants reported knowing three people who died by suicide, with a greater perceived impact of the recent suicide death.

#### 3.1.2 Key Outcome Variables

On average, at T1 for both groups, the mean scores of psychological distress were considered high, and the mean scores of complicated grief, resilience, and professional help‐seeking intentions were above mid‐point, respectively. The mean scores of suicidal ideation reflected high rates of suicidal distress, although typically below severe risk for suicidal behavior.

Independent samples *t*‐tests were conducted to examine significant differences in key outcome variables between the BCI and active control groups. Results, summarized in Table 2, showed no statistically significant group differences in any of the key outcomes at T1.

**TABLE 2:** Summary of means and standard deviations (SD) of key outcome variables for total sample and mean differences between BCI and active control groups at T1.

Outcome variables	Total sample (*n* = 99)	BCI group (*n* = 52)	Active control group (*n* = 47)	*t*(df)
	M (SD)	M (SD)	M (SD)	
Psychological distress	28.03 (8.95)	28.47 (8.34)	27.55 (9.66)	−0.50 (91)
Missing: *n* (%)	6 (6.1%)	3 (5.8%)	3 (6.4%)	[Cohen's *d* = −0.10]
Suicidal ideation	7.71 (10.95)	8.50 (10.64)	5.10 (8.39)	−1.66 (86)
Missing: *n* (%)	11 (11.1%)	6 (11.5%)	5 (10.6%)	[Cohen's *d* = −0.35]
Complicated grief	40.27 (16.23)	39.50 (14.59)	41.26 (18.31)	0.47 (76)
Missing: *n* (%)	21 (21.2%)	8 (15.4%)	13 (27.7%)	[Cohen's *d* = 0.11]
Resilience	42.74 (7.03)	42.00 (6.80)	43.63 (7.27)	1.12 (92)
Missing: *n* (%)	5 (5.1%)	1 (1.9%)	4 (8.5%)	[Cohen's *d* = 0.23]
Professional help‐seeking intentions	5.34 (1.40)	5.39 (1.20)	5.26 (1.63)	−0.42 (83)
Missing: *n* (%)	14 (14.1%)	5 (9.6%)	9 (19.1%)	[Cohen's *d* = −0.09]

### 3.2 Intervention Effectiveness

Mixed model ANOVAs were conducted to examine group differences in key outcome measures. Results, summarized in Table 3, showed no statistically significant changes in any outcomes from T1 to T2.

**TABLE 3:** Summary of group differences in key study outcomes.

Outcomes	BCI group		Active control group		*F*	Partial eta squared
	T1, M (SD)	T2, M (SD)	T1, M (SD)	T2, M (SD)		
	**(*n* = 14)**		**(*n* = 16)**			
Psychological distress	28.07 (7.76)	27.00 (8.15)	29.13 (10.71)	27.56 (8.93)	0.11 (1, 28), *p* = 0.741	< 0.01
	**(*n* = 10)**		**(*n* = 10)**			
Suicidal ideation	8.00 (6.88)	11.00 (11.76)	3.60 (5.02)	3.90 (5.02)	0.66 (1, 18), *p* = 0.426	0.04
	**(*n* = 12)**		**(*n* = 12)**			
Complicated grief	46.08 (11.58)	45.08 (19.98)	37.92 (16.32)	34.42 (14.91)	0.38 (1, 22), *p* = 0.544	0.02
	**(*n* = 15)**		**(*n* = 15)**			
Resilience	43.73 (4.35)	44.53 (6.98)	43.13 (10.45)	41.93 (8.99)	0.99 (1, 28), *p* = 0.331	0.03
	**(*n* = 12)**		**(*n* = 13)**			
Professional help‐seeking	5.71 (1.14)	5.38 (1.15)	5.08 (1.35)	5.19 (1.28)	0.63 (1, 23), *p* = 0.435	0.03

### 3.3 Webpage Traffic: Access of Information

Table 4 shows that the most frequently viewed webpage across groups was the page about “grief about suicide” (*n* = 81). Overall, the BCI group accessed the webpages more frequently than the active control group; with at least double the views for every webpage except one (“Accessing crisis support”). The “Accessing professional support” page was accessed ten times more by BCI participants than those in the active control group. Average time spent on the pages across groups was short (range = 6 s to 4:29 min). Time spent on the pages was generally similar across groups except for the “Practical challenges associated with bereavement” page for which the active control group spent longer on (4:29 min vs. 2:49 min) while the BCI group spent longer on the “Accessing professional support page” (2:33 min vs. 36 s). An additional page was available on the website “I need help now” to ensure participants from either group were able to easily access information if they were in imminent danger or crisis. Throughout the duration of the study, this page was accessed 12 times by participants in the BCI group and eight times by the active control group.

**TABLE 4:** Webpage access by BCI and active control group participants.

Webpage content	BCI group		Active control group	
	Page views	Average time spent on page (min:s)	Page views	Average time spent on page (min:s)
Message 1: Grief after suicide	63	02:13	18	01:58
Message 2: Practical challenges associated with bereavement	24	02:49	8	04:29
Message 3: Navigating relationships after suicide	10	00:13	5	00:24
Message 4: Accessing crisis support	6	00:24	5	00:21
Message 5: Looking after yourself	8	00:06	4	00:20
Message 6: Accessing peer support	7	00:11	4	00:31
Message 7: Accessing professional support	20	02:33	2	00:36

### 3.4 Intervention Feedback

Twelve participants provided feedback about the BCI on the T2 survey (see Table 5). On average, these participants indicated that the intervention was enjoyable, helpful, easy to understand, interesting, useful, and worth recommending to others.

**TABLE 5:** Feedback from BCI group regarding intervention acceptability.

Intervention feedback		*n* = 12	
	**None, *n* (%)**	**Some, *n* (%)**	**All, *n* (%)**
I received the texts	0 (0%)	0 (0%)	12 (100%)
I read the text messages	0 (0%)	1 (8.33%)	11 (91.66%)
I found the text messages interesting	0 (0%)	4 (36.36%)	7 (63.64%)
I found the text messages useful	0 (0%)	6 (54.55%)	5 (45.45%)
I found the text messages relevant	1 (9.09%)	4 (36.36%)	6 (54.55%)
I found the text messages helpful	1 (8.33%)	4 (33.33%)	7 (58.33%)

Rated on a scale of 1–5 (strongly disagree‐ strongly agree)	Disagree, *n* (%)	Neutral, *n* (%)	Agree, *n* (%)
I liked the text messages	1 (8.33%)	3 (25.00%)	8 (66.66%)
I would recommend this intervention to others who have lost a person to suicide	1 (8.33%)	1 (8.33%)	9 (83.33%)

Rated on scale of 1–10 (completely disagree‐ completely agree)	Mean (SD) [range]
I enjoyed the intervention	6.73 (2.05) [2–9]
I found the intervention helpful	6.92 (1.98) [2–9]
The intervention was easy to understand	9.27 (1.10) [7–10]
I found the intervention to be interesting	7.83 (2.59) [2–10]
I would use the intervention in the future	7.58 (2.39) [2–10]
I would recommend the intervention to other people who might benefit from it	8.17 (1.40) [6–10]
The skills I learned from the intervention helped me a lot in everyday life	5.67 (2.53) [1–9]
*What did you like about the text messages?*	*What did you dislike about the text messages?*
“consistent and varied (in terms of topics)” “I like that it reminded me that it is a difficult time and reminded me to be human” “it felt like somebody was checking in on me” “nice to know people care” “small pieces of accessible information I could choose how much I wanted to engage with” “strategies” “that they came. I really like receiving them” “that you don't have to talk to anyone”	“could have been more. Plus the early ones weren't appropriate considering the time my daughter has been gone” “I thought they were bland” “it didn't feel like it was meant for me, as my family member who suicided had long term mental health challenges and made multiple suicide attempts over the decade preceding his death, so there was a sense of relief at his death that then triggered guilt” “not enough no contact information, I don't want to talk” “not much [I didn't like], liked overall” “that the messages came from different numbers and sometimes they were just too blah if you know what I mean. It's such a complex topic and some of the messages just felt like they were a brush stroke but didn't go into any depth”
*How could the text messages be improved?*	
“intervention could go for longer. I found that it was helpful for as long as it did go; however, I certainly noticed when it stopped” “make more acknowledgement that for some people the suicide was a shock but for others there may have been previous attempts. Also, some people, particularly caring for someone with mental health challenges, might have complex relationships with the deceased” “more practical advice needed, maybe a checklist of things to do, like eat shower” “more tailored messages” “sending memes/uplifting messages” “short daily ones would be great. Or at least more often” “content relevancy to be matched to different people and their suicide issues”	

### 3.5 Intervention Feedback: Interviews

Of the eleven participants who consented to an interview, eight BCI participants completed a feedback interview, while three were unavailable at the scheduled or rescheduled time. De‐identified quotes are used to illustrate the nature of the feedback, along with the corresponding participant interview numbers.

#### 3.5.1 Content Was Experienced as Relevant and Validating

Participants reported that receiving the messages validated their bereavement experience (P2, P3, P8), for example, “my experiences are probably the same as other people's going through this situation” (P8) and the messages were “sitting there on my phone ready to go, you know, I really appreciated it” (P1) and were appropriate in length “bite chunks as opposed to just this overwhelming…booklet that you read” (P3). Practical information (relating to the funeral process, coronial processes and financial matters) was viewed as particularly helpful (P2, P5, P7); as was information about relationships, the impact of suicide bereavement (P2, P7) and coping and help‐seeking mechanisms (P6). Participants described the language and tone of the text messages as appropriate and none of the BCI content was identified as upsetting or triggering (P5, P8). One participant, however, found the format lacking in human connection and reported this was the reason for withdrawing from the study “I found them to be clearly an automated response” (P7).

#### 3.5.2 Proactive Information Sharing Was Key, but Timing Was Important

Participants expressed that receiving the BCI meant they did not have to go and start searching for information themselves (P1, P8) as it was provided to them, as a main advantage. The text messages were described as proactive in nature—one participant referred to the messages as “something that was reaching out to me but also something I can do on my end” (P8). Even for those who had researched or had navigated bereavement before (P1, P2), the delivery of the intervention through text messages was reported as beneficial. The BCI was described as a helpful additional layer of knowledge (P1, P2). For example, one participant with good knowledge of bereavement concepts stated it was “nice to have prompts to remind me” (P2). Half of the participants reported that the messages connected them with relevant support services.

Reflections on the timing of the intervention were mixed. For some, messages were delivered too soon after the death when they were “still in shock” (P7), or in “the wrong place” (P1). Others stated that because they did not sign up immediately after the death, the messages were irrelevant to their current stage of grief, especially for those that had already navigated the practical rituals associated with a death (e.g., funeral); noting that text messages would have been more useful earlier on, albeit no perfect timing is possible given the uniqueness of each grief experience (P2, P8). Despite this, participants described that they were able to re‐read text messages (P1, P3, P8) providing additional reflection opportunities.

Finally, mixed experiences about the duration of the intervention were described. For some, text messages ended too soon (P1, P4), with only one participant finding the length adequate, “I think that was completely fine like the length of it was also good. Because I remember like once I started to feel better it was coming to close to the end of this study” (P8).

#### 3.5.3 Tailoring Message Content to Improve Relevance and Impact

Participants expressed their support of the scale‐up of this intervention “The idea of sending people those messages is a good one” (P6), and valued receiving the intervention, “I just think it's really valuable. I'm really glad you're doing it, and I just really want to encourage you, to be supportive as possible yeah” (P2).

Participants suggested tailored messages, as P6 expressed “Sort of personalise that information a little bit based on what a person needs.” Such personalisation would need to consider the death information, and time of day the message was received (P6, P8) but also geographic location and access (or lack thereof) to further support services being offered from the embedded links in the messages. Most reported a preference to receive messages as early as possible in the bereavement experience (P2, P3, P5, P6, P7). Participants suggested that ideally messages would be received soon after a death and recommended working with First Responders or the Coroner's Office (P1, P3, P7) to enhance dissemination.

## 4 Discussion

Many current suicide postvention interventions lack evidence of effectiveness. Evidence that does exist shows promise for therapeutic and educational support provided by trained facilitators (Andriessen, Krysinska, Hill, et al. 2019). However, such support will not always be accessible due to geographic isolation, the individual's financial constraints, and/or the limited accessibility of service providers. Innovation that provides accessibility at a low cost is required. The current study presents a novel postvention response, as the first study to develop an SMS‐based BCI with co‐designed messages to support people in the community following suicide bereavement. The acceptability of this BCI is promising, although the effectiveness of the intervention compared to psychoeducation alone could not be established. Vulnerability of those who have been bereaved by suicide, and the continuing need to identify effective ways of delivering support, highlight a need for future work.

### 4.1 The Suicide Bereaved Are Living With Significant Mental Health Impacts

These findings were consistent with prior larger sample and population evidence identifying significant psychological distress and complicated grief, including suicidal thoughts, for those bereaved by suicide (Johnson et al. 2010; Braun and Clarke 2006), with over half of this sample reporting suicidal thoughts in the past year (ABS 2022). This is considerably higher than 15.3% of the general community (ABS 2022). The proportion of study participants who lived in regional, rural, and remote areas was more than double that of population norms (60% vs. 28%, respectively) (AIHW 2024), suggesting that this type of intervention may be suitable when accessing services due to geographic isolation and distance to physical services (AIHW 2024).

### 4.2 We Were Unable to Demonstrate the Effectiveness of an SMS‐Based BCI for People Bereaved by Suicide

This study was unable to demonstrate the effectiveness of a BCI for people bereaved by suicide as an added benefit over standard psychoeducation. The BCI and active control groups did not show statistically significant changes in key outcomes over the study period. A lack of statistically significant effect is not unusual in efficacy studies for people who have been bereaved (McGill, Bhullar, Pearce, et al. 2022; Andriessen, Krysinska, Kõlves, and Reavley 2019). Typically, postvention interventions are more likely to be associated with positive outcomes. Intervention effects can vary over time and across different outcomes (e.g., grief vs. depression) (Andriessen, Krysinska, Hill, et al. 2019; Andriessen, Krysinska, Kõlves, and Reavley 2019). This may signal underpowered effects due to the small sample sizes at follow‐up. The lack of effect could also indicate that the intervention alone was not sufficient to result in improvements to wellbeing. Within this context, it would be appropriate to consider how the BCI could be delivered as part of a larger postvention service system.

### 4.3 SMS‐Based BCI Was Generally Well‐Received and Valued, but Could Be Improved

For those participants who responded at T2, feedback about the BCI format and content was generally positive with participants. Easy access to relevant information and supports, at the “right” time during the bereavement process, is consistently identified as important by people bereaved by suicide (Hofmann et al. 2025) however, it is a key challenge for those delivering services to those bereaved by suicide (Finlayson‐Short et al. 2020; Ligier et al. 2020; McKinnon and Chonody 2014; Peters et al. 2016). SMS BCI may be an acceptable way of addressing this issue. Tailoring information to a person's situation and information needs, including content, style, and timing of message delivery, was deemed important. Reflecting on a targeted approach, the scope to tailor content to an individual's needs and distress may improve recruitment and engagement with digital mental health interventions, potentially enhancing the effectiveness of such interventions (Carrandi et al. 2023) and the integration of services. The low retention rates suggest that future BCIs may need to better address important implementation barriers, such as tailoring content to increase relevance and timeliness, and accounting for individual preferences regarding the depth and type of content delivered. A platform specifically designed for the BCI delivery rather than the survey platform used for this pilot (Qualtrics) would allow for personalized messaging (e.g., using the individual's name) and integration with a postvention service would address some of the issues identified by participants in relation to personalized tailoring to individual circumstance and time since death.

### 4.4 Strengths and Limitations

This study has a number of strengths, including the RCT design, with only 5% (*n* = 24) of primary studies on suicide bereavement and postvention using a control/intervention study design (Maple, Pearce, et al. 2017). The content of the BCI messages was co‐designed with bereaved people, postvention workers, and researchers from across the globe (McGill, Bhullar, Batterham, et al. 2022), which is the current best practice in suicide research. Furthermore, the mixed‐methods design provided scope to explore the context to better understand support needs and how the intervention can be improved. Limitations of the study included significant recruitment and retention issues, which limited our ability to determine the effectiveness of the intervention in terms of health outcomes. The study was scheduled to commence in 2020; however, due to the global COVID‐19 pandemic and the unknown impact this would have on participation and general levels of distress, the trial was postponed for over a year, with resource constraints restricting recruitment to less than half of the target sample. The low numbers recruited may have been an artifact of the conditions of the pandemic or may reflect implementation barriers for these types of low‐intensity interventions, particularly when people bereaved by suicide see a need for more comprehensive, therapeutic, or personalized interventions. Regardless of the reasons for lower‐than‐expected demand for the intervention, the small sample size limited our ability to perform between‐group analysis with adequate power. One participant opted out of receiving the text messages.

Our qualitative results are further limited to a small subset of participants and may be biased toward those who received the intervention positively. This study also had a high proportion of female participants and a small representation of culturally and linguistically diverse people. Additional future work would be required to assess the suitability potential adaptation an intervention like this among highly exposed vulnerable population groups, including cultural (e.g., First Nations people) or occupational (e.g., military, first responders) groups. Finally, our study included participants who self‐identified as bereaved by suicide in the last year. However, many identified multiple suicide exposures, some of which are likely to have occurred earlier (more than 12 months before participating in the study), and for which we have no information about supports or resources accessed for these other exposures. This may have limited this study's ability to detect effects in the context of participants' existing experience and knowledge. Future research could assess time since bereavement and prior exposure to bereavement information and resources to enable personalized message tailoring to bereaved individuals' needs and time since the death of their person(s).

### 4.5 Building the Effectiveness Evidence Base

This study highlights a number of considerations for future research. While the estimated sample size was intended to account for a 50% drop‐out rate, the actual retention rate was even lower (32%). Although the ongoing impacts of the pandemic are likely to have played a role in retention, the initial low estimate also highlights the need to consider how participation can be supported throughout the lifetime of a distally delivered clinical trial. Capturing information about a variety of key outcomes must be balanced with the need to keep questionnaire batteries nondemanding. Data linkage (e.g., with routinely collected support service access data) may offer an alternative approach to broadening the understanding of outcomes in a manner that does not increase participant burden. For BCI studies, there may also be specific challenges in maintaining investment in the research process over time, as the intervention is automated and requires no direct contact with research staff. There may also be opportunities for SMS BCI studies specifically. For example, linking receipt of the message with invitations to provide immediate feedback on the message's relevance, timing, and impact may lead to higher engagement over time. It could provide an option to offer additional support that “reaches out”, based on participants' responses during real‐time feedback processes.

## 5 Conclusions

Our findings suggest that recently bereaved people valued this easy‐to‐deliver, low‐intensity suicide postvention BCI; however, a well‐powered study is still required to understand the effectiveness of this approach. Future trials of this SMS‐based BCI could consider tailoring messages to meet individuals' needs and embedding the intervention within a broader system of support. Working more specifically with service providers within the postvention space may offer a way of broadening the reach of the intervention and building the evidence base for meeting the needs of people bereaved by suicide.

## Author Contributions

**Myfanwy Maple:** conceptualization, formal Analysis, funding acquisition, methodology, project administration, supervision, writing – original draft, investigation, writing – review and editing. **Navjot Bhullar:** conceptualization, formal analysis, funding acquisition, methodology, supervision, writing – original draft, investigation, writing – review and editing. **Philip J. Batterham:** conceptualization, formal analysis, funding acquisition, methodology, writing – original draft, investigation, writing – review and editing. **Katie McGill:** data curation, formal analysis, project administration, writing – original draft, investigation, writing – review and editing. **Sarah Wayland:** data curation, formal analysis, writing – original draft, investigation, writing – review and editing. **Alayna Carrandi:** data curation, formal analysis, project administration, writing – original draft, investigation, writing – review and editing.

## Ethics Statement

Ethics approval was granted by the University of New England's Human Research Ethics Committee (HE21‐175).

## Consent

Participants consented to participate in the study and agreed that their anonymous research data (including quotes) may be published.

## Conflicts of Interest

Myfanwy Maple is Deputy Chair of Suicide Prevention Australia Research Fund Advisory Committee. She had no influence in the funding of this project.

## Data Availability

The data that support the findings of this study are available on request from the corresponding author. The data are not publicly available due to privacy or ethical restrictions.

## Appendix A

### SMS Text Message Content

**Table:**

Welcome message	Hello, [Name]. Welcome to the SignPost study. You will receive a series of text messages from us over the next 6 weeks. Remember, crisis services are always available. Lifeline‐ 13 11 14; Suicide Call Back Service‐ 1300 659 467 If you would like to find out more information about the study, please go to [Webpage]. We hope you find the messages useful and look forward to your feedback. Warm regards, SignPost Research Team. Opt out of future text messages here: [link]
Message 1	Hi, [Name]. It's the SignPost Team here. Grief affects everyone differently. Feelings can be intense and unpredictable. You may feel shocked, angry, guilty, numb, relieved, despairing or even as if everything is not real. Whatever feelings you are having are ok and they can change over time. If you would like to find out more about grief after suicide, please go to: [Grief after suicide webpage] Sometimes talking to someone about how you are feeling can be helpful. Information about relevant services are here: [Webpage]. If you need crisis support call Lifeline on 13 11 14 Opt out of future text messages here: [link]
Message 2	Hi, [Name]. It's the SignPost Team here. There are many practical challenges that come up after a person has died by suicide. You are often dealing with unfamiliar and confusing legal and financial processes that are difficult to navigate. If you would like some help with challenges, such as the coroners' process, finances and supporting your children while grieving, please follow this link: [Webpage]. If you need crisis support call Lifeline on 13 11 14 Opt out of future text messages here: [link]
Message 3	Hi, [Name]. It's the SignPost Team here. Sometimes relationships with others change after a person has died by suicide. There are many reasons why. Sometimes, people grieve differently, are unkind or insensitive or others can withdraw or avoid contact because they do not know what to say. Sometimes relationships grow stronger after a death. Having relationships change at the same time as living with grief is hard. Remember, if something isn't working for you, it is ok to tell the other person or to limit contact. If you would like more information about relationships after suicide, please go to [Webpage]. If you need crisis support call Lifeline on 13 11 14 Opt out of future text messages here: [link]
Message 4	Hi, [Name]. It's the SignPost Team here. Sometimes after a person has died by suicide, you may experience extremely low moods or may even have thoughts about suicide. This is not uncommon. If this happens for you, it is important to let others know. You are not alone and there is help available. Talking to a trusted person, a GP, Lifeline or the Suicide Call Back Service are all good places to start. Lifeline‐ 13 11 14; Suicide Call Back Service‐ 1300 659 467 Follow this link for more information: [Webpage] Opt out of future text messages here: [link]
Message 5	Hi, [Name]. It's the SignPost Team here. After losing a person to suicide, resuming routines and returning to work or school can be challenging. You may feel unable to keep up with doing the things that keep you well. Taking care of yourself and your well‐being is extremely important and even little things can make a difference. Things like taking time out for yourself, staying connected with people you care about, trying to eat well, staying hydrated, exercising and not using drugs and alcohol can all be helpful. For some other ideas, follow this link: [Webpage]. If you need crisis support call Lifeline on 13 11 14 Opt out of future text messages here: [link]
Message 6	Hi, [Name]. It's the SignPost Team here. It can feel very lonely after a suicide. Many people find it helpful to hear from and talk with others who have been through similar experiences. To hear from others or to find out more about connecting with others who have lost a person to suicide, follow this link: [Webpage]. If you need crisis support call Lifeline on 13 11 14 Opt out of future text messages here: [link]
Message 7	Hi, [Name]. It's the SignPost Team here. There is no right way to grieve and different things work for different people. Some people find it useful to talk with others. Often this is with family and friends. Sometimes it is also helpful to talk with someone who is not connected to your social world. This is where peer and professional support can help. For information on the different professional supports available, follow this link: [Webpage]. If you need crisis support call Lifeline on 13 11 14 Opt out of future text messages here: [link]
Ending message	Hi, [Name]. It's the SignPost Team here. Thank you for your participation in the SignPost study. This is the end of the text message intervention. You will shortly receive a link to the feedback survey which will help us understand whether the text messages were helpful or not and where you can let us know what you thought of them. Remember, if it would help to talk to someone about how you are traveling, there are support services available. Lifeline‐ 13 11 14; Beyond Blue‐ 1300 22 4636. We appreciate your time in supporting this project and look forward to your feedback. Warm regards, The SignPost Research Team.
